# Induction of Ras by SAF-1/MAZ through a feed-forward loop promotes angiogenesis in breast cancer

**DOI:** 10.1002/cam4.362

**Published:** 2014-11-30

**Authors:** Alpana Ray, Bimal K Ray

**Affiliations:** Department of Veterinary Pathobiology, University of MissouriColumbia, Missouri

**Keywords:** Angiogenesis, breast cancer, Ras, SAF-1, VEGF expression

## Abstract

In the majority of breast cancers, overexpression and hyperactivation of Ras in the tumor microenvironment play significant role in promoting cancer cell growth, angiogenesis, and metastasis. We have previously shown that vascular endothelial growth factor (VEGF) expression in triple negative breast cancer cells is regulated, at least in part, by SAF-1 (serum amyloid A activating factor 1) transcription factor. In this study we show that transformation of normal MCF-10A breast epithelial cells by constitutively active, oncogenic Ras, induces the DNA-binding activity and transcription function of SAF-1. Furthermore, we show that inhibition of MEK/MAPK-signaling pathway prevents Ras-mediated activation of SAF-1. Interestingly, silencing of SAF-1 expression in breast cancer cells by SAF-1-specific short hairpin RNAs (shRNAs) significantly reduced H-Ras and K-Ras mRNA level. We show that SAF-1 is a direct transcriptional regulator of *H-Ras* and *K-Ras* and overexpression of SAF-1 increases *H-Ras* and *K-Ras* gene expression. Chromatin immunoprecipitation (ChIP) analyses demonstrated in vivo interaction of SAF-1 at highly purine-rich sequences present at the proximal promoter region, upstream of the transcription start site, in *H-Ras* and *K-Ras* genes. Previous studies have shown that these sequences are nuclease hypersensitive and capable of forming G4 quadruplex structure. Together, our results show the presence of a novel transactivating loop, in which, Ras and SAF-1 are interconnected. These findings will help defining molecular mechanisms of abnormal overexpression of Ras in breast tumors, which seldom show genetic *Ras* mutations.

## Introduction

Tumor microenvironment (TME) is a dynamic entity which determines tumor growth, invasion, and metastasis. A number of cellular processes regulate different elements of the TME and determine the status of the tumor ranging from dormancy to aggressive growth. Angiogenesis is one of these distinct elements that trigger tumor growth by promoting vascular endothelial growth factor (VEGF) expression and subsequent vascular growth 1,2. In the TME, activated Ras proteins have been shown to play an important role in VEGF synthesis by the tumor cells and this finding was further validated by the ectopic expression of a constitutively active mutant *Ras* plasmid DNA which has been shown to directly promote VEGF expression and in vitro angiogenesis 3–5. In correlation, farnesyltransferase inhibitors that prevent posttranslational modification and activation of Ras proteins have been shown to inhibit VEGF expression 6 and its secretion from tumor cells 7. Ras-mediated VEGF synthesis has implicated Raf-1/MEK/MAPK and PI3K/Akt pathways in the activation of several transcription factors including Sp1, AP2, and HIF-1/2*α* in the induction of VEGF expression 8–10. Sp1 has been shown to induce VEGF in an HIF-1-independent mechanism 11 and a strong association between Sp1 and gastric cancer has been reported 12. Interestingly, the Sp1-binding sites in VEGF promoter is not restricted for Sp1 alone as other transcription factors with similar DNA-binding motif, namely, Cys_2_-His_2_ type zinc finger, often bind to this promoter region 13.

Recently, we reported a novel mechanism of VEGF induction in aggressive triple negative breast cancer cells by transcription factor serum amyloid A activating factor 1 (SAF-1) 14 which belongs to the family of Cys_2_-His_2_ type zinc finger and binds to the *VEGF* proximal promoter region that overlaps the Sp1-binding site. SAF-1 and its human homolog, myc-associated zinc finger protein (MAZ) 15 have been shown to be overexpressed in many human cancers 14,16–19. Marked increase of MAZ mRNA is observed at the terminal phase of human chronic myelogenous leukemia (CML) and an increased transcription from the *MAZ* gene has been closely linked to the malignant phenotype of this disease 20. SAF-1/MAZ is reported to be activated via phosphorylation by various protein kinases. Previous reports have indicated that in response to various inflammatory stimuli, DNA binding and transcriptional function of SAF-1/MAZ are further increased via its phosphorylation by various protein kinases, including MAPK, PKC, PKA, and CK2 21–24. These studies, however, did not address whether oncogenic signaling could activate SAF-1/MAZ in the TME. Since SAF-1/MAZ is highly induced and activated in breast cancer cells and breast tumors 14 and Ras-mediated signaling pathway is highly prevalent in these tumor cells, raises the possibility of this pathway in SAF-1/MAZ activation. In the present study, we demonstrate that in breast cancer cells, constitutively activated, oncogenic *ras* increases DNA-binding and transcriptional activities of SAF-1/MAZ via phosphorylation through the MAP-kinase pathway. In this context, SAF-1/MAZ could be a target for Ras/MAPK-directed therapeutics.

Human *Ras* family contains three genes (*H-, N-,* and *K-Ras*), which encode four highly homologous Ras proteins of ∽21 kDa size. *H-Ras* and *K-Ras* genes were first identified as cellular homologs of Harvey and Kirsten rat sarcoma virus oncogenes while *N-Ras* was identified in human Neuroblastoma cells 25. The Ras proteins are guanine-binding proteins and involved in relaying various extracellular signals to cytoplasm via GTP/GDP on-off switching 26. In normal cells, Ras proteins are only transiently activated, which then act on multiple downstream effector pathways to regulate cell proliferation, survival, and differentiation. Mutations of the *Ras* family of proto-oncogenes are very common; being found in 20–30% of many human cancers, including bladder, pancreas, colon, thyroid, and lung 27. However, breast cancer has very low, <5%, incidence of *Ras* gene mutations 27. Oncogenic mutations in *ras* genes confer gain-of-function by providing constitutive activation that drives uncontrolled cell proliferation and malignant transformation. Besides mutation, overexpression of *Ras* that facilitates Ras-mediated signaling has been reported in human cancer 28–30. Surprisingly, the mechanisms regulating how overexpression of *Ras* occurs are less understood. During our studies on Ras-mediated SAF-1 induction, we have observed that once activated, SAF-1 acts as a transcriptional inducer of *H-Ras* and *K-Ras* and we reveal the presence of a transactivation loop in which oncogenic Ras increases DNA-binding and transcriptional activities of SAF-1 which in turn, increases transcription of *Ras* forming a feed-forward regulatory pathway.

## Material and Methods

### Cell lines

MCF-10A and MDA-MB-468 cells were obtained from American Type Culture Collection (ATCC), cultured and stored following ATCC protocol of authentication by short terminal repeat analysis. The cells were maintained in Dulbecco's modified Eagle's medium containing high glucose (4.5 g/L) and supplemented with 7% fetal bovine serum.

### Establishment of cell lines and transfection assay

To generate MCF-10A-*ras* cells, MCF-10A cells were transfected with pCMV*ras*V12 plasmid, expressing constitutively active *ras* oncogene (Clontech Laboratories, Mountain View, CA, USA). As a control, MCF-10A cells were transfected with the same vector containing only the *neo* gene. The transformed cells were selected by culturing in medium containing 400 *μ*g/mL geneticin (G418). In some transfection assays, SAF-1/MAZ short hairpin RNAs (shRNAs), and control shRNAs (Santa Cruz Biotechnologies, Dallas, TX, USA) were used. For overexpression of SAF-1, cells were transfected with either pcD-SAF-1 expression plasmid or a mutant SAF-1 plasmid construct. For transfection analysis, the cells were transfected with different reporter plasmid DNA (0.5 *μ*g) as described before 14 and cell extracts were used chroramphenicol acetyl transferase (CAT) assay as described before 14.

### Reporter gene and expression plasmids

Wild-type and mutant SAF-CAT reporter plasmids, designated as wt SAF-CAT and mt SAF-CAT, respectively, were constructed by ligating into the pBLCAT2 vector, three tandem copies of the wild-type SAF-binding element of SAA gene promoter from nucleotide position −254 to −226 containing the following sequences: wild-type: 5′-CCCTTCCTCTCCACCCACAGCCCCCATGG-3′ and mutant: 5′-CAATGAGTCGAGACCGTCGACATCCATGG-3′ as described earlier 31. 1.2 VEGF-CAT reporter plasmid, that contains human *VEGF* promoter sequences from nucleotide position −1179 to +21, was prepared by ligating this DNA into pBLCAT3 vector. A tandem copy of two SAF-1-binding sites is located within nucleotide position −130 and −30 of this *VEGF* promoter DNA 14. 1.2 Δ(−130/−30) VEGF-CAT reporter plasmid, containing a deletion of DNA sequences from −130 to −30 of *VEGF* promoter, was prepared as described earlier 14 by ligating polymerase chain reaction (PCR)-generated fragments of *VEGF* promoter DNA into pBLCAT3 vector. The 0.6H-Ras-CAT reporter was prepared by ligating, into pBLCAT3 vector, a promoter region of human Ha-*ras* 32, from nucleotide position −552 to +28 that corresponds to the junction of the noncoding first exon called exon 0 and intron 1. Similarly, the 0.68K-Ras-CAT reporter plasmid was prepared by ligating, in pBLCAT3 vector, a promoter region of human Ki-*ras* 33, from nucleotide position −465 to +215 that corresponds to the junction between noncoding exon 0 and intron 1. Both, *H-Ras* and *K-Ras* promoter DNA fragments were prepared by PCR amplification and verified by DNA sequencing. SAF-1 expression plasmid, pcD-SAF-1, was prepared by inserting full-length SAF-1 cDNA with a FLAG tag into pcDNA3 vector (Invitrogen Corporation, Grand Island, NY, USA). A mutant SAF-1, mut pcD-SAF-1(V71), containing a mutation at the MAP kinase phosphorylation site by replacement of threonine at amino acid position 71 of SAF-1 with valine was used. Both the wild-type pcD-SAF-1 and mutant pcD-SAF-1(V71) were described earlier 21.

### Preparation of nuclear extract and DNA-binding assay

Nuclear extract preparation and DNA-binding assays were performed as described before 14. As ^32^P-labeled probes, sequences from −135 to +29 of *VEGF* promoter that contains SAF-1-binding elements 14 and a SAF-1-binding oligonucleotide of *SAA* promoter as described before 14 were used. In some binding reactions, antibody (Ab) against SAF-1 and Sp1 or normal IgG (Santa Cruz Biotechnology) were added to the reaction mixtures during a preincubation period of 30 min on ice. As a competitor of SAF-1 binding, some binding reactions contained 50 pmol of a canonical SAF-binding double-stranded oligonucleotide having an upper strand sequence of 5′-CCCTTCCTCTCCACCC-3′. As a nonspecific oligonucleotide competitor, a random sequence of oligonucleotide with an upper strand sequence of 5′-TCGAACTGAACTTGAG-3′ was used.

### RNA isolation and qRT-PCR

Total RNA was isolated using a RNA isolation kit (Qiagen, Germantown, MD, USA). Relative expression levels of SAF-1, H-Ras, and K-Ras was determined by real-time RT-PCR using gene-specific primers. The cDNAs were prepared by reverse transcription from 0.5 *μ*g of total RNA using TaqMan reverse transcription reagents and analyzed for SAF-1, Ha-Ras, K-Ras, and glyceraldehyde-3-phosphate (GAPDH) according to the manufacturer's protocol (Applied Biosystems, Life Technologies, Grand Island, NY, USA). Transcript levels were normalized against GAPDH. All experiments were done using biological triplicates and experimental duplicates. The primers were: forward, 5′-GCTCAGGACTTAGCAAGAAG-3′ and reverse, 5′-GTATTTACATAATTACACACTTTG-3′ for human K-Ras; forward, 5′-GGGGCAGTCGCGCCTGTGAA-3′ and reverse, 5′-CCGGCGCCCACCACCACCAG-3′ for human H-Ras; forward, 5′-CTCCAGTCCCGCTTCT-3′ and reverse, 5′-GGGAGCAAGTCCACCT-3′ for human SAF-1/MAZ; forward, 5′-CCCTTCATTGACCTCAACTACATG-3′ and reverse, 5′-TGGGATTTCCATTGATGACAAGC-3′ for human GAPDH.

### ChIP assay

Chromatin immunoprecipitation (ChIP) assays were performed following a method as described 14 with minor modifications. Cells grown in culture were cross-linked with 1% formaldehyde for 10 min followed by addition of 0.125 mol/L glycine for 5 min and washed in PBS buffer. Following lysis of the cells and sonication, DNA-protein complexes in the lysates were subjected to immunoprecipitation using anti-SAF-1 or normal IgG. After precipitation of the immunocomplex with protein G-agarose, followed by washing and extraction with elution buffer, immunoprecipitated DNA was used as template in PCR with specific primers spanning the target region of human *K-ras* and human *H-ras* gene promoters. Primers used for amplification of human *K-Ras* promoter were 5′-TCCGGGTCAGAATTGGCG-3′ and 5′-GTTCCGCGCTCGATTCTTCT-3′, which yields an amplicon of 313-bp. Primers used for amplification of human *H-Ras* promoter were 5′-GTCTCCAGCCAAGCCCAAC-3′ and 5′-ACTCACCGTTCACAGGCG-3′, which yields an amplicon of 363-bp.

### Statistics

To compare multiple sets of data, a one-way analysis of variance (ANOVA) with post hoc Fisher's least significant difference test was used. For paired data sets, a two-tailed *t* test was used. Values of *P *< 0.05 were considered to represent a significant difference.

## Results

### SAF-1/MAZ DNA-binding and transcriptional activities are induced in oncogenic rasV12 transformed MCF-10A cells

**Figure 1:**
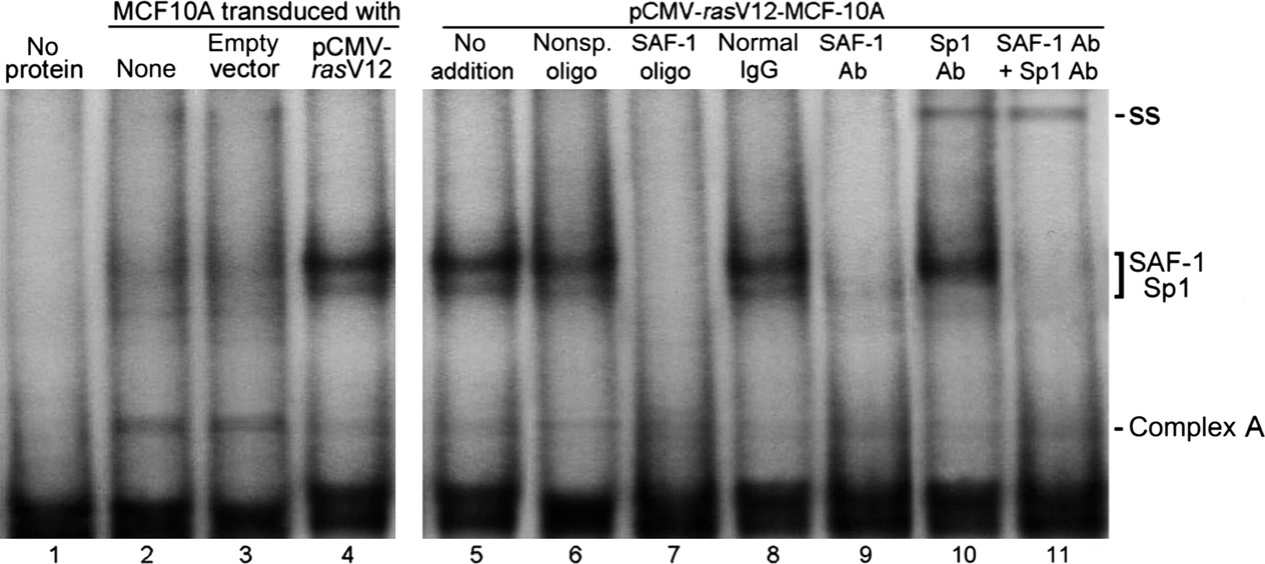
Increase of SAF-1 DNA-binding activity in oncogenic *ras*-transformed MCF-10A cells. Nuclear extracts (10 *μ*g of protein) prepared from untreated (lane 2), empty vector transformed (lane 3) or pCMV-*ras*V12 transformed (lanes 4–11) MCF-10A cells, as indicated, were incubated with ^32^P-labeled *VEGF* DNA (−135 to +29) containing SAF-1-binding element. Lane 1 contains no nuclear extract. Resulting DNA-protein complexes were fractionated in a 6% nondenaturing polyacrylamide gel. In some assays, 50-fold molar excess of either nonspecific oligonucleotide (lane 6) or SAF-1-binding competitor oligonucleotide (lane 7) or normal IgG (lane 8) or antibody to SAF-1 (lanes 9 and 11) or antibody to Sp1 (lanes 10 and 11) were included during a preincubation reaction. Migration positions of SAF-1-specific complex are indicated. Super-shift (ss) of Sp1-specific DNA-protein complex is indicated. Complex A represents a DNA-protein complex present in normal MCF-10A cell nuclear extract is not affected by either SAF-1 competitor oligonucleotide, or the antibodies. SAF-1, serum amyloid A activating factor 1; VEGF, vascular endothelial growth factor.

To understand whether in the TME, SAF-1/MAZ activity is increased by oncogenic signaling, we stably transduced MCF-10A cells, which has a low basal SAF-1/MAZ activity 34, with a constitutively active oncogenic pCMV*ras*V12 expression vector or a control empty vector DNA. MCF-10A is a spontaneously immortalized cell line originated from nonmalignant breast epithelial cells and these cells have many characteristics of normal breast epithelium, including inability to form colonies in soft agar and tumors in nude mice 35. Previous studies have shown that upon the introduction of *Ras* oncogene, MCF-10A cells undergo transformation and these transformed cells exhibit hall marks of transformation and epithelial-mesenchymal transition (EMT), including anchorage-independent growth, loss of requirement of hormones, invasiveness, and tumorigenicity in nude mice 36,37. As shown in Figure1, DNA-binding activity of SAF-1 was low in untransfected or empty vector transfected MCF-10A cells, but in oncogenic pCMV*ras*V12 transfected MCF-10A cells, the DNA-binding activity became markedly higher (Fig.1, lanes 2–4). Presence of SAF-1 in this DNA-protein complex was verified by using molar excess of competitor SAF-1-binding oligonucleotide and an anti-SAF-1 antibody, both of which inhibited the DNA-protein complex formation (Fig.1, lanes 7 and 9). Involvement of Sp1 transcription factor in this DNA-protein complex was evident by the appearance of a super-shifted complex (ss) when anti-Sp1 antibody was used (Fig.1, lanes 10 and 11). It is likely that SAF-1 and Sp1 together bind to the *VEGF* promoter. Indeed, such interaction of SAF-1 and Sp1 may have a synergistic effect on the VEGF expression as these two transcription factors have earlier been shown to synergize in the transcriptional induction process 38. A minor DNA-protein complex, complex A, was not affected by either SAF-1 or Sp1-specific antibodies and is composed of KLF-4 as described earlier 34. Ras expression seems to have little effect in this complex formation.

**Figure 2:**
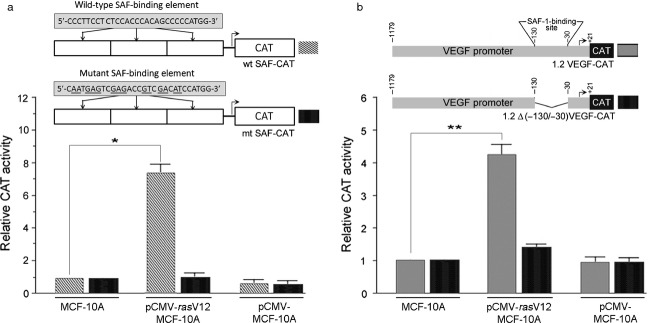
Induction of SAF-1 promoter function in oncogenic *ras*-transformed MCF-10A cells. (A) MCF-10A or pCMV-*ras*V12 or empty vector, pCMV, transformed MCF-10A cells were transfected with 1.0 *μ*g each of wild type (wt) or mutant (mt) SAF-CAT reporter construct. The wild-type SAF-CAT reporter contains three tandem repeats of SAF-1-binding elements ligated in pBLCAT2 vector. The mutant SAF-CAT contains three tandem copies of mutant SAF-1-binding elements ligated to pBLCAT2. Relative CAT activity in ras- and empty vector-transformed cells was determined by comparing it to that in normal MCF-10A cells. Results represent an average of three separate experiments. *P <* *0.05. (B) The same three sets of cells, as those in (A), were transfected with 1.0 *μ*g each of 1.2 VEGF-CAT containing a 1200 base pair *VEGF* promoter with a SAF-1-binding site located within nucleotide position −130 and −30 or a deletion mutant of *VEGF* promoter lacking the SAF-1-binding site and ligated in pBLCAT3 vector. Relative CAT activity in ras- and empty vector-transformed cells was determined by comparing it to that in normal MCF-10A cells. Results represent an average of three separate experiments. **P <* *0.02. SAF-1, serum amyloid A activating factor 1; CAT, chroramphenicol acetyl transferase; VEGF, vascular endothelial growth factor.

To assess whether increased DNA-binding activity of SAF-1 in Ras-transformed MCF-10A-*ras* cells leads to an alteration in its transcriptional function, we evaluated SAF-1-driven promoter activity using a CAT reporter gene that contains three tandem copies of a bona fide SAF-1-binding element 31. Transcription from this promoter was evaluated by transfection of this reporter plasmid in MCF-10A, MCF-10A-*ras*, or empty vector transformed MCF-10A cells (Fig.2A). The CAT expression was significantly higher in Ras-transformed MCF-10A cells as compared to untransformed or empty vector transformed cells (Fig.2A). The reporter gene containing mutated SAF-binding element, mt SAF-CAT, showed no response to oncogenic *ras*, indicating that Ras-activated SAF-1 that binds to a bona fide SAF-1-binding element can drive CAT expression. To assess whether Ras-activated SAF-1 can promote angiogenesis-associated gene promoter activity, we used *VEGF* promoter-driven CAT reporter system (Fig.2B). This assay revealed that CAT activity is highly increased in *ras*-transformed MCF-10A-*ras* cells that are transfected with the 1.2 VEGF-CAT plasmid. This region of *VEGF* promoter contains a SAF-1-binding site with two tandem SAF-binding elements located between nucleotide position −130 and −30 14. Involvement of Ras-activated SAF-1 in the promoter activation was further verified when the *VEGF* promoter with a deletion in the SAF-1-binding site, failed to show a significant induction in the ras-transformed cells (Fig.2B). Together, these results suggested that Ras increases both the DNA-binding activity and transcription function of SAF-1.

### Ras activates SAF-1/MAZ activity through ERK/MAPK-signaling

**Figure 3:**
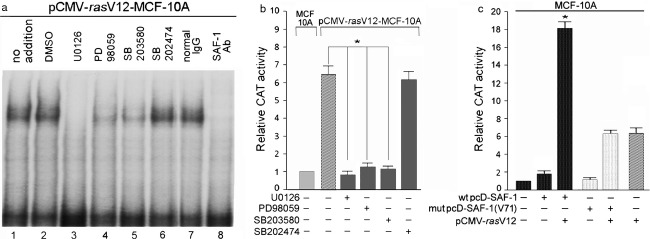
Oncogenic *ras*-mediated activation of SAF-1 is regulated via MEK/MAPK pathway. (A) Oncogenic pCMV-*ras*V12 transformed MCF-10A cells were either left untreated or incubated with DMSO (vehicle), MEK 1/2 inhibitor U0126 (20 *μ*mol/L), MAP kinase inhibitors PD98059 (20 *μ*mol/L), SB203580 (20 *μ*mol/L), or SB202474 (20 *μ*mol/L) for 24 h. Nuclear extracts (10 *μ*g of protein) prepared from these cells, as indicated, were incubated with ^32^P-labeled DNA containing SAF-1-binding element of the SAA promoter. Resulting DNA-protein complexes were fractionated in a 6% nondenaturing polyacrylamide gel. (B) MCF-10A or pCMV-*ras*V12-transformed MCF-10A cells were transfected with equal amount (1.0 *μ*g) of wt SAF-CAT reporter plasmid. Cells were grown for an additional 24 h in absence or presence of 20 *μ*mol/L each of U0126, PD98059, SB203580, or SB202474, as indicated. Relative CAT activity in ras-transformed cells was determined by comparing it to that in normal MCF-10A cells. Results represent an average of three separate experiments. *P <* *0.05. (C) MCF-10A cells were transfected with equal amount (1.0 *μ*g) of wt SAF-CAT reporter plasmid. In addition, some cells were cotransfected with either a wild-type SAF-1 expression plasmid, wt pcD-SAF-1 (0.5 *μ*g) or a mutant SAF-1 expression plasmid, mut pcD-SAF-1(V71) (0.5 *μ*g). Also, some cells were transfected with Ras expression plasmid, pCMV*ras*V12 (0.5 *μ*g), as indicated. Relative CAT activity was determined by measuring CAT activity of cotransfected MCF-10A cells compared to that of SAF-CAT transfected MCF-10A cells. Results represent an average of three separate experiments. *P <* *0.05. SAF-1, serum amyloid A activating factor 1; CAT, chroramphenicol acetyl transferase.

The *Ras* oncogenes utilize various signaling pathways to modify downstream effectors that drive tumorigenesis. Among these pathways, the Raf-MEK-ERK-MAPK cascade is a major one. Raf serine/threonine kinases (c-Raf-1, A-Raf, and B-Raf) phosphorylate and activate MEK1/2 dual specificity kinases, which then phosphorylate and activate the ERK1 and ERK2 MAPKs. Activated ERKs translocate to the nucleus where they phosphorylate and regulate various transcription factors. To determine if Ras-mediated activation of SAF-1 proceeds through Raf-MEK-ERK-MAPK cascade, MCF-10A-*ras* cells were grown with or without PD98059, which is a specific inhibitor of mammalian MEK-1/2 and ERK (P42/44 MAP kinase). PD98059 markedly inhibited DNA-binding activity of SAF-1/MAZ in MCF-10A-*ras* cells (Fig.3A). U0126, a specific inhibitor of MEK1/2 and SB203580 which inhibits both p42/44 and p38 MAP kinases also inhibited the binding activity. Identity of the protein in the DNA-protein complex was verified as SAF-1 by the ablation of the complex by anti SAF-1 antibody (Fig.3A, lane 8).

To test whether changes in the DNA-binding activity of SAF-1 in response to the MEK and MAPK inhibitors also affects its transcriptional function, we used CAT reporter assay using wt SAF-CAT, where CAT expression is driven by the SAF-binding promoter element (Fig.3B). Treatment of the CAT vector-transfected cells with U0126, PD98059, and SB203580 but not by SB202474, which is an inactive analog of SB203580, significantly reduced the ras-mediated induction of SAF-1/MAZ function, suggesting involvement of MEK-ERK-MAPK cascade in the activation of SAF-1/MAZ by Ras.

To examine whether SAF-1/MAZ is directly targeted by Ras, we used a mutant SAF-1 construct, SAF-1(V71), that contains a defective MAP-kinase phosphorylation site 21. The DNA-binding activity and transactivation potential of this mutated SAF-1 protein has earlier been shown to be markedly less than that of wt SAF-1 21. We cotrasnfected MCF-10A cells with the reporter plasmid wt SAF-CAT along with oncogenic pCMV*ras*V12 and wild-type pcD-SAF-1 or mutant pcD-SAF-1(V71) plasmid DNAs (Fig.3C). There was marked increase in the reporter gene expression in the cells cotransfected with wild-type pcD-SAF-1 and oncogenic pCMV*ras*V12 as compared to the cells cotransfected with mutant pcD-SAF-1(V71) and oncogenic pCMV*ras*V12 plasmid DNAs (Fig.3C). The level of induction in cells transfected with mutant pcD-SAF-1(V71) + pCMV*ras*V12 was same as that with pCMV*ras*V12 alone. This level of induction is the result of Ras-mediated activation of endogenous SAF-1 in the MCF-10A cells. These data suggested that MAPK site of SAF-1 is necessary for Ras-mediated activation of SAF-1 and confirmed the involvement of MEK-ERK-MAPK-signaling in *Ras*-mediated activation of SAF-1/MAZ.

### Knockdown of SAF-1/MAZ in breast cancer cells represses Ras gene expression

**Figure 4:**
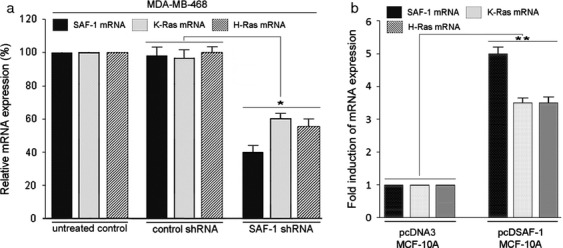
Silencing of SAF-1/MAZ reduces mRNA level of K-Ras and H-Ras. (A) MDA-MB-468 breast cancer cells were transfected with MAZ/SAF-1 short hairpin RNA (shRNA) lentiviral particles, control shRNA lentiviral particles (Santa Cruz Biotechnology), or left untreated. Total RNA isolated from these cells was subjected to qRT-PCR analysis with primers specific for SAF-1/MAZ, K-Ras and H-Ras. The results were normalized to the level of GAPDH in each sample and represent an average of three separate experiments. *P <* *0.05. (B) MCF-10A cell were transfected with pcDNA3 empty vector or pcD-SAF-1 expression plasmid DNAs, as indicated. Total RNA isolated from these cells was subjected to qRT-PCR analysis with primers specific for SAF-1/MAZ, K-Ras, and H-Ras, as described in (A). The results were normalized to the level of GAPDH in each sample and represent an average of three separate experiments. **P <* *0.03. SAF-1, serum amyloid A activating factor 1; qRT-PCR, quantitative reverse transcription-polymerase chain reaction.

To further validate the role of Ras in SAF-1 activation, we knocked-down SAF-1 in the breast cancer cells. The evidences that overexpression of SAF-1/MAZ occurs in many human cancers and SAF-1/MAZ transcription factor is a target of activated Ras proteins, raised the questions, what downstream pathways are affected during aberrant activation of SAF-1 in cancer. MDA-MB-468 breast cancer cells are triple negative and contain no mutation in *Ras* genes 39. Also, presence of high level of SAF-1/MAZ in MDA-MB-468 cells 14 renders it suitable to assess the effect of silencing SAF-1/MAZ. To examine, SAF-1/MAZ expression was silenced in MDA-MB-468 cells by transfection of SAF-1/MAZ shRNA and global gene expression profile was examined. These data revealed down-regulation of several prominent cancer-linked genes that included MMP-1, MMP-9, MMP-14, p21^WAF1/CIP1^, hTERT, PPARg1, Prox-1 and VEGF (data not shown), and surprisingly, it showed downregulation of Ras mRNA expression. Quantitative RT-PCR analysis revealed downregulation of both H-Ras and K-Ras mRNAs by about 50% in SAF-1 knockdown MDA-MB-468 cells (Fig.4A). Reciprocally, overexpression of SAF-1 in MCF-10A cells that normally contain low level of SAF-1 34 and therefore suitable to examine the effect of overexpression of SAF-1, showed increased H-Ras and K-Ras mRNA levels by more than threefold compared to empty vector transfected cells (Fig.4B).

### SAF-1 acts as a transcriptional regulator of Ras

**Figure 5:**
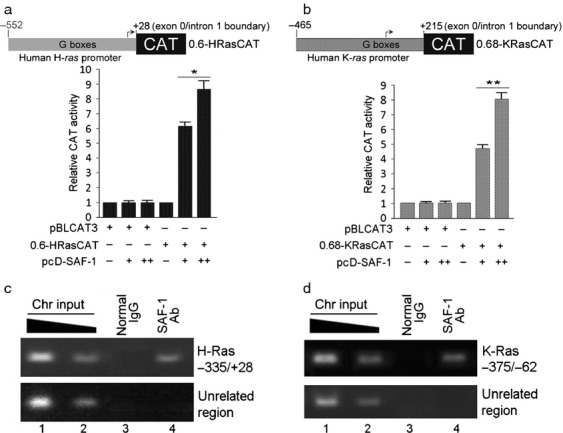
SAF-1 increases *H-Ras* and *K-Ras* promoter function by binding to the G-box promoter elements. MDA-MB-468 cells were cotransfected with equal amount (1.0 *μ*g) of pBLCAT3 or 0.6HRas-CAT reporter plasmid DNA (A) or 0.68KRas-CAT reporter plasmid DNA (B), as indicated, and increasing concentration (0.5 and 1.0 *μ*g) of pcD-SAF-1 plasmid DNA. Relative CAT activity was determined by comparing the CAT activities of transfected plasmids with that of pBLCAT3 alone. (C and D). MDA-MB-468 cells were cross-linked with formaldehyde and chromatin isolated from these cells was subjected to ChIP analysis by immunoprecipitating with anti-SAF-1 antibody or control IgG, as indicated. The precipitated chromatin DNA or input DNA was used for PCR amplification using *H-Ras* (C) and *K-Ras* (D) gene-specific primers. An unrelated upstream region was amplified to serve as negative control. SAF-1, serum amyloid A activating factor 1; CAT, chroramphenicol acetyl transferase; CHIP, chromatin immunoprecipitation; PCR, polymerase chain reaction.

A strong correlation between SAF-1 and Ras, in vivo, suggested that SAF-1 could be involved in regulating *Ras* gene expression. It has long been known that human breast tumors have low percentage of activating *Ras* mutations 27,28 but that does not necessarily mean that Ras plays no role in breast cancer. In fact there is extensive experimental evidence demonstrating overexpression of wild-type Ras proteins and high level of activated Ras proteins in human breast tumor tissues 29,30, suggesting activation of upstream mechanisms regulating Ras in breast cancer. Overexpression of Ras proteins is also very common in other human cancers that harbor genetic *Ras* mutations. Thus, upregulation of *Ras* may be one mechanism leading to carcinogenic transformation in many types of human cancer. To determine, if SAF-1 is a direct transcriptional regulator of *Ras*, we examined the promoter function of human *H-Ras* and *K-Ras* genes in SAF-1 overexpressing cells. It is worthy of mention that like the protein coding regions, the proximal promoter region of human *Ras* genes are highly similar. The expression 0.6HRas-CAT and 0.68KRas-CAT reporter genes was consistently increased, in a dose-dependent manner, in pcD-SAF-1 expression plasmid transfected cells (Fig.5A and B). These data suggested that SAF-1 is a transcriptional regulator of human *H-Ras* and *K-Ras* genes.

### SAF-1 interacts at a purine-rich region in the proximal promoters of H-Ras and K-Ras

The proximal promoter regions of the three functional *Ras*-genes are highly similar and contain many copies of the GGGC/A/TGGG element (G-boxes) or its inverted complement, which have been shown to be essential for the promoter transcriptional activity 40. SAF-1/MAZ also binds to highly purine-rich sequences and the G-boxes in *Ras* promoters appeared to bear considerable similarity with the consensus SAF-1/MAZ -binding element, RGGGRAGGRR, in which R is a purine 31. We employed ChIP analysis, which readily detects in vivo interaction of a transcription factor with the DNA in the chromosomal context. Formalin-fixed and SAF-1 antibody immunoprecipitated chromatin from MDA-MB-468 breast cancer cells showed specific enrichment of the purine-rich promoter regions of H-*ras* and K-*ras* (Fig.5C and D). There was no enrichment of these sequences when a nonspecific antibody was used, indicating specificity of SAF-1 interaction. Together these results demonstrated that SAF-1 interacts with *Ras*genes in vivo and is capable of inducing expression of H-*Ras* and K-*Ras* genes.

## Discussion

**Figure 6:**
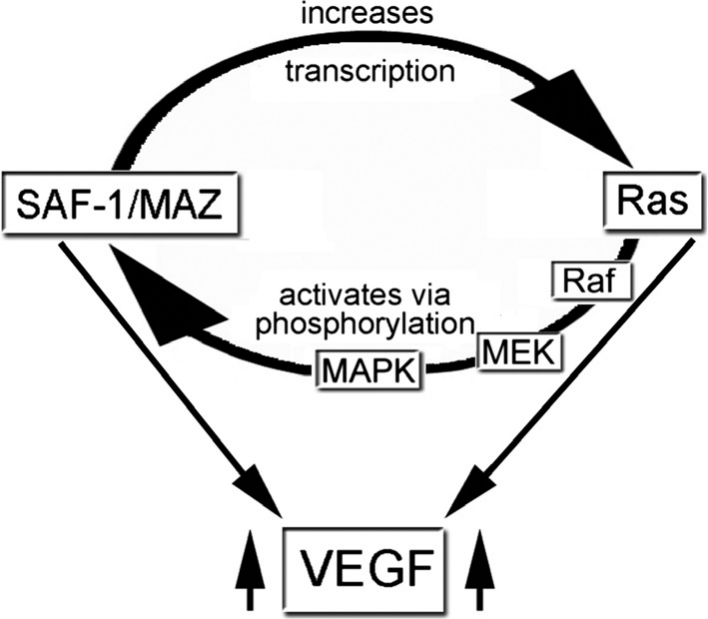
Model illustrating the Ras→SAF-1/MAZ→VEGF signaling network axis. Ras activates SAF-1/MAZ by Raf-MEK-ERK-MAPK pathway of phosphorylation. Activated SAF-1/MAZ promotes Ras expression by transcriptional induction of *ras* gene. The cyclical feed-forward regulation of SAF-1/MAZ and Ras promotes angiogenesis by promoting VEGF expression 14,27. SAF-1, serum amyloid A activating factor 1; VEGF, vascular endothelial growth factor.

Angiogenesis is crucial for tumor cell growth and proliferation for which cancer cells employ multiple pathways to increase *VEGF* transcription. Identification of novel mechanisms of VEGF-mediated angiogenesis in the context of cancer biology is therefore very important for improving cancer therapy. We provide evidence and a model (Fig.6) illustrating how oncogenic, activated Ras can increase the DNA-binding and transcription function of SAF-1/MAZ transcription factor, a transcriptional regulator of VEGF. In turn, high level of SAF-1/MAZ, that is present in the breast cancer cells, increases *Ras* gene expression allowing more SAF-1/MAZ activation by activating MAP kinase pathway. These findings provide an explanation for persistently high level of expression of Ras proteins in many types of human cancer. In this context, SAF-1/MAZ could be a target for Ras/Raf/MAPK-directed therapeutics.

SAF-1 transcription factor originally was isolated as an inflammation-responsive, Cys_2_-His_2_-type zinc finger protein 31. Its human homolog MAZ was identified as a regulator of *c-myc* gene 15. Regulation of SAF-1 activity is mediated via phosphorylation of this protein by various protein kinases, which markedly increase its DNA-binding activity 21–24. In this paper, we show for the first time, that Ras is involved in the activation of SAF-1 and utilizes MAPK-signaling pathway. Based on the results obtained from the use of a series of inhibitors, namely U0126, PD98059, and SB22025 that inhibit the MEK-MAPK-signaling, it was evident that Ras-mediated activation of SAF-1 proceeds through modulation of the Raf-MEK-ERK-MAPK signaling cascade. In the Ras-Raf-MEK-ERK-MAPK cascade Ras binds to and promote activation of Raf and activated Raf, in turn, phosphorylates and activates the mitogen-activated protein kinase/extracellular signal-regulated kinase (ERK) kinase MEK1 and MEK2 dual specificity kinases. MEKs then phosphorylate and activate the ERK1 and ERK2 mitogen activated protein kinases. It is noteworthy that the role of PI3K-Akt kinase signaling in mediating Ras-mediated activation of SAF-1 has not been examined and awaits further investigation.

We showed that silencing of SAF-1 reduces K-Ras and H-Ras mRNA level (Fig.4) and SAF-1 is a direct transcriptional regulator of these two *Ras* genes (Fig.5A and B). These results were confirmed by in vivo ChIP assays, which demonstrated that SAF-1 interacts at the G-rich sequences that are present upstream of the transcription start site in these two *Ras* promoters (Fig.5C and D). Like the protein coding sequences, the proximal promoters of three human *Ras* genes are highly similar and conserved and contain highly G-rich sequences, including multiple repeats of GGGCGGG and its inverted complements. Guanine-rich nucleic acid sequences are known to self-associate and form unusual structures known as G-quartets, G-4 DNA, or G-quadruplexes. G-quadruplexes have been shown to be present in regions of biological significance, such as human telomeres and in the promoter regions of several cancer-linked genes, including *c-myc*, *bcl-2*, *c-kit*, *VEGF*, *Ras*, *HIF-1α*, and *Rb* 41,42. The G-rich sequences that are capable of forming G-quadruplexes are not rigid and can somewhat vary. For example, in human telomere, tandem repeats of the hexanucleotide d(TTAGGG)_n_ form G-quadruplexes 43, while the G-quadruplexes in gene promoters are composed of G-tracts with unequal numbers of guanines and various numbers of intervening bases 44. Among these, the G_3_NG_3_ motif appears to be widespread and evolutionarily selected to serve as a stable core in promoter intramolecular sequences of G-quadruplex structure. In correlation with our observation, Pur-1, the mouse homolog of SAF-1, is found to interact with G-quartets in the insulin-linked polymorphic region (ILPR, also known as IDDM2), located in the promoter region of the human insulin gene 45, while MAZ, the human homolog of SAF-1 is shown to interact with quadruplex forming GA-element in *K-Ras* promoter 46. The SAF-1-binding element in the *VEGF* promoter 14 also forms a G-quadruplex structure 47. Our finding of the G-rich sequences and a possible G-quadruplex structure in *Ras* gene promoter as a site of interaction of SAF-1/MAZ and involvement of such interaction in *ras* expression could have a significant impact in understanding the role of SAF-1/MAZ in breast cancer and perhaps in other cancer as well due to ubiquitous presence of Ras in many cancers and its direct link to SAF-1/MAZ which is revealed in the current study.

A common feature of transcription networks is the presence of feedback regulatory loops used for amplification of an initial signal through positive reinforcement or through a negative regulation. Our investigation elucidated an important aspect of cancer cell signaling that cancer thrives on multiple intermolecular conjunctions to sustain oncogenicity by depicting (Ras→SAF-1/MAZ→VEGF) signaling network axis (Fig.6). Downregulation of Ras mRNAs in SAF-1 silenced cells further showed the range of downstream networks that can be affected during aberrant silencing or overexpression of a transcription factor. In this context, a number of clinical trials have been aimed at disrupting Ras signal transduction and inhibiting VEGF function, independently. Inhibitors of farnesylation that disrupt activation of Ras were not very efficient because of specificity of the transfer of farnesyl/geranyl moiety 48,49. Similarly, VEGF inhibitors although prolonged survival rate in cancer patients, also caused increase in the red blood cells. As inhibition of SAF-1 can suppress Ras and VEGF expression, these findings offer a new possibility for cancer therapy by targeting SAF-1/MAZ function that may provide an improved treatment option for cancer. Targeting SAF-1/MAZ can also benefit other diseases that are linked with aberrant expression of VEGF, including arthritis, defective wound repair, endometriosis, and blindness.
